# Selective Expansion of Tregs Using the IL-2 Cytokine Antibody Complex Does Not Reverse Established Alopecia Areata in C3H/HeJ Mice

**DOI:** 10.3389/fimmu.2022.874778

**Published:** 2022-06-15

**Authors:** Eunjin Lee, Mingyu Kim, You Jeong Lee

**Affiliations:** ^1^ Department of Life Sciences, Pohang University of Science and Technology (POSTECH), Pohang, South Korea; ^2^ Graduate School of Medical Science and Engineering, Korea Advanced Institute of Science and Technology (KAIST), Daejeon, South Korea; ^3^ Research Institute of Pharmaceutical Sciences, College of Pharmacy, Seoul National University, Seoul, South Korea

**Keywords:** alopecia areata (AA), Treg, IL-2, anagen, hair

## Abstract

Alopecia areata (AA) is an autoimmune disease mediated by NKG2D-expressing cytotoxic T lymphocytes destroying hair follicles in the skin. It is one of the most common autoimmune diseases, but there is no effective treatment modality approved by the FDA. Regulatory T cells (Tregs) are crucial for suppressing autoreactive T cells, and in the skin, they promote hair growth by inducing anagen. Based on this, we tested the therapeutic potential of expanded Tregs in AA using the C3H/HeJ mouse model. In mice with AA, NKG2D-expressing CD8 T cells widely infiltrate both haired and hairless skin areas, which have tissue-resident memory T-cell phenotypes. Tregs in the skin express CD25, CTLA-4, GATA-3, and Jagged1 and efficiently proliferate with IL-2 cytokine antibody complex. However, expanding Tregs in the skin did not induce anagen in normal mice, indicating that they are necessary but not sufficient for anagen induction. Also, they fail to suppress autoreactive CD8 T cells in the skin to reverse established AA in C3H/HeJ mice. These results suggest that Treg expansion alone is not sufficient for AA treatment, and combined immunotherapy is required.

## Introduction

Alopecia areata (AA) is the most frequent cause of inflammation-induced hair loss affecting 0.1%–0.2% of the general population at any given point in time, with an estimated lifetime risk of 2.1% (1, 2). It is mediated by NKG2D-expressing autoreactive cytotoxic T lymphocytes (CTL), which destroy hair follicles (HFs), leading to transient or permanent hair loss. Genetic and environmental factors provoke the collapse of immune privilege in HFs, and other stressors induce the activation of NKG2D+ CTL in the skin and its draining lymph nodes (LNs) (3). Activated CTLs express high levels of IFN-γ, which upregulates MHC I and II expressions on HF stem cells and induce the presentation of follicle-associated auto-antigens (4). RNA profiling revealed that AA lesions in mice and humans contain increased levels of granzymes A and B and cytokine IL-15, and IL-15 can effectively increase granzyme B expression in auto-reactive T cells (5). However, blocking these cytokines after disease onset has no therapeutic effects and there is no effective treatment modality that can permanently reverse this process (5).

Janus kinase (JAK)/signal transducer and activator of transcription protein (STAT) signaling mediates various cellular events, including cytokine signaling cascade of lymphocytes and quiescent status of HF stem cells (6). Strikingly, Jak inhibitors (JAKis) could reverse established AA in C3H/HeJ mice and human patients (5, 7), and its derivatives are under clinical trials. However, JAKis increase sensitivity to infection and cancer by nonspecific suppression of immune cell activation. Also, human patients invariably relapse upon treatment cessation, indicating that its therapeutic effect is transient (8–10).

Regulatory T cells (Tregs) suppress the activation of autoreactive T cells, and their therapeutic potential to treat autoimmune disease has been successfully tested in several autoimmune diseases, including inflammatory bowel diseases (IBDs) and type I diabetes (T1D) (11–14). Interestingly, Tregs in the skin promote hair growth by stimulating the proliferation of HF stem cells through the expression of Notch ligand family member, Jagged1 (15). Based on these findings, we hypothesized that Treg expansion in the skin could be a treatment modality of AA by inhibiting HF-reactive CTLs and facilitating hair growth by anagen induction. To address this issue, we set up the C3H/HeJ mouse model of AA as previously described (16). In this mouse, we found that NKG2D+ CTLs in the AA lesion have the tissue-resident memory T-cell phenotype. To expand Tregs in the skin, we used intradermal (ID) injection of IL-2/anti-IL-2 antibody complex (IL-2c), which induces selective expansion of Tregs expressing Jagged1, CTLA-4, and GITR. However, although Tregs expanded about 8- to 10-fold in the skin, it was not sufficient to induce anagen in normal mice. In mice with AA, expanded Tregs also failed to decrease the number of pathogenic CD8 T cells and resume hair growth. These results indicate that expanded Tregs have limited therapeutic potential in AA, unlike other autoimmune diseases.

## Materials and Methods

### Mice

C57BL/6 and C3H/HeJ mice were purchased from the Jackson Laboratory and maintained under specific pathogen-free conditions at POSTECH. Experiments were performed in compliance with institutional guidelines as approved by the Institutional Animal Care and Use Committee of POSTECH (2013–01–0012).

### Animal Model of Alopecia Areata

AA mice were generated as previously described (16). Briefly, SDLNs were removed from mice that spontaneously developed AA, and cells were cultured with AR10 (advanced RPMI 1640, Gibco) containing 10% FBS (Atlas Biologicals), 2 mM GlutaMAX (Welgene), and 100 U/ml penicillin–streptomycin (Welgene) supplemented with IL-2, IL-7, and IL-15. Cells were stimulated with anti-CD3 and CD28-coated microbeads (Dynabeads, Thermofisher) and intradermally transferred to at least 10-week-old normal-haired C3H/HeJ mice during the second telogen phase.

### Single-Cell Isolation

To make a single-cell suspension of mouse skin, minced skin tissue was incubated in RPMI media containing 10% FBS, 1% HEPES, and 100 U/ml penicillin–streptomycin (Welgene) supplemented with 2 mg/ml collagenase D (Roche), 0.1 mg/ml DNase I (Biosesang), and 0.5 mg/ml hyaluronidase (Biosesang) at 37°C for 50 min. Then, an additional 20 ml of media was added and shaken by hand for 30–45 s, and the cell suspension was filtered through a sterile 40-μl cell strainer into a new 50-ml conical tube. The cell suspension was then pelleted and re-suspended in PBS for cell counting and staining. Perfused lung tissues were harvested, minced, and digested in 5 ml of RPMI media containing 2 mg/ml collagenase D and 0.1 mg/ml DNase I on a shaker at 37°C for 45 min followed by filtration through a 70-μm strainer. Mononuclear cells were obtained after 40% and 70% Percoll (Merck) gradient centrifugation at 2,000 rpm for 20 min at RT. Liver tissue was minced and filtered through a 70-μm strainer, and mononuclear cells were isolated after 40% and 70% Percoll gradient centrifugation.

### Immunofluorescence

Skin tissue was fixed in 4% paraformaldehyde (PFA, Electron Microscopic Science) for 1 h, washed with PBS, and left in 30% sucrose overnight before embedding in OCT. Tissue sections were stained primarily with anti-CD3-BV480 (1:1,000, 17A2), anti-CD4-AF488 (1:250, RM4-5), anti-CD8a-PE (1:250, 53-6.7), anti-FoxP3-APC (1:250, FJK-16s), and anti-GL3-biotin (1:250, GL3), followed by secondary SA-AF750 (1:10,000) at RT. Slides were then washed in PBS and mounted with DAPI-containing medium. Images were obtained using Leica DM6B with the THUNDER system.

### IL-2 Treatment

Human IL-2 (hIL-2, GenScript), anti-hIL-2 antibody (BD, clone 5344.111), and mouse IL-2 Fc (absolute antibody) were used for Treg expansion. The IL-2 cytokine antibody complex (IL-2c) was made by mixing 1 μg of hIL-2 and 5 μg of 5344.111 per 18 g of mice weight.

### Local Treatment of Ruxolitinib

The affected dorsal skin of C3H/HeJ mice with AA was treated daily for 12 weeks with ruxolitinib dissolved in 10% DMSO and mixed with Aquaphor as 0.5% ointment as described previously (17).

### Anagen Induction

The dorsal hair of 60-day-old C57BL/6 mice was shaved with a clipper before the treatment. Control mice were treated with topical 10% DMSO (vehicle control) or 2% ruxolitinib (APExBIO), as described previously (7). IL-2c was IP injected for three consecutive days, and hair growth was monitored until day 80.

### *Ex Vivo* Expansion of Treg

*Ex vivo* expansion of Treg was performed as previously described (18). Briefly, spleens of Thy1.1^+^ C57BL/6 mice were single-cell isolated and enriched for CD4 T cells by using the CD4 T cell isolation kit (Miltenyi Biotec). CD25^+^ cells were isolated using the FACSAria II cell sorter (BD Biosciences). After checking the purity (≥95%), Tregs were cultured in RPMI media containing 10% FBS, 100 U/ml penicillin–streptomycin (Welgene), 1× GlutaMAX-1, 1 mM sodium pyruvate, 10 mM HEPES, 1× nonessential amino acids, 10 μM 2-mercaptoethanol, and 2,000 IU/ml of recombinant human IL-2 (hIL-2, GenScript). Tregs were seeded into 12-well plates with anti-CD3 and CD28-coated microbeads (Dynabeads, Thermofisher) at a 2:1 cell-to-bead ratio. On days 2, 4, 6, 9, and 11, culture volume was doubled by adding hIL-2 supplemented fresh medium.

### Flow Cytometry

Isolated mononuclear cells were stained with surface markers for 30 min at 4°C, and dead cells were excluded by staining Zombie Aqua Fixable viability dye (BioLegend). For intracellular staining, surface-stained cells were fixed and permeabilized with the Foxp3/transcription factor staining buffer set (Thermo Fisher Scientific). The following fluorescent dye-labeled antibodies were purchased from BD Biosciences, BioLegend, and eBioscience: anti-CD4-BUV395 (1:400, GK1.5), anti-CD25-APC (1:400, PC61), anti-CD314 (NKG2D)-PE (1:200, CX5), anti-CD62L-PerCP-Cy5.5 (1:400, MEL-14), anti-CD103-APC (1:400), anti-CD44-AF700 (1:400, IM7), anti-TCRβ-APC-Cy7 (1:400, H57-597), anti-CD45.2-BV605 (1:200, 104), anti-CD8-BV650 (1:400, 53-6.7), anti-CD11b-BV711 (1:1,000, M1/70), anti-B220-BV711 (1:400, RA3-6B2), anti-CD357 (GITR)-PE-Cy7 (1:400, DTA-1), anti-CD339 (Jagged1)-PE (1:100, HMJ1-29), anti-Eomes-AF488 (1:200, Dan11mag), anti-T-bet-PE-Cy7 (1:400, 4B10), anti-FoxP3-PE-CF594 (1:400, MF23), anti-GATA3-PE (1:400, TWAJ), and anti-CD152 (CTLA-4)-PE-Texas Red (1:400, UC10-4F10-11). Stained cells were analyzed using BD LSR Fortessa, and data were analyzed using Flow Jo software (Tree Star).

### Statistical Analysis

Statistical analyses were performed with Prism software (GraphPad). *p*-values were calculated using a two-tailed unpaired Student’s *t*-test.

## Results

### Tissue-Resident Memory CD8 T Cells Infiltrate in Skin Lesion of C3H/HeJ Mice With AA

**Figure 1:**
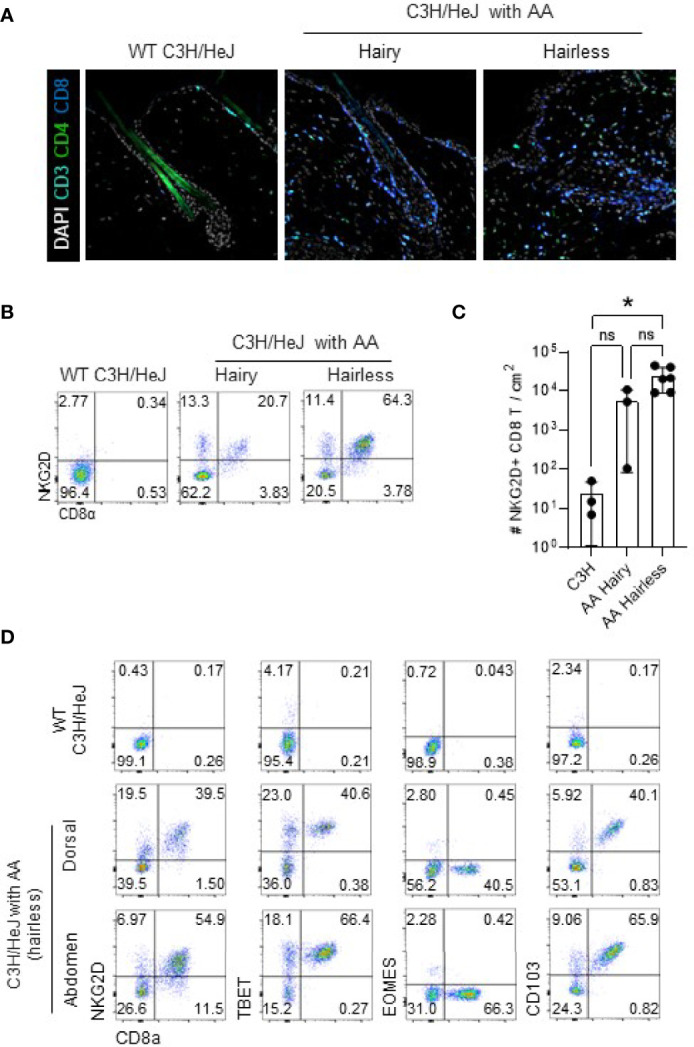
NKG2D^+^ TBET^+^ Eomes¯ CD103^+^ CTL cells infiltrate lesional and non-lesional skin of AA. **(A)** Representative immunofluorescence images show CD8 and CD4 T cells around the HF in the dorsal skin of indicated mice. **(B, C)** Representative dot plots show NKG2D^+^ CD8 T cells in the dorsal skin of indicated mice after gating on live CD45^+^CD19¯CD11b¯ cells **(B)**. Graph shows statistical analysis in normal C3H/HeJ mice (*n* = 3) and hairy (*n* = 3) and hairless (*n* = 6) skin of C3H/HeJ mice with AA. **(D)** Representative dot plots show expression of NKG2D, TBET, Eomes, and CD103 after gating on live CD45^+^CD19¯CD11b¯ cells in indicated mice. Representative results from more than three independent experiments are shown. Horizontal bars indicate mean values, error bars show SD, and each dot represents an individual mouse **(C)**. An unpaired two-tailed *t*-test was used. *p < 0.05. CTL, cytotoxic T lymphocytes; HF, hair follicle. ns, non-significant.

We generated a mouse model of AA by transferring cultured skin-draining LN cells obtained from C3H/HeJ mice that spontaneously developed AA to normal-haired C3H/HeJ mice as previously described (16) (**Figure S1**). Immunofluorescence staining of hairless skin lesions shows a dense infiltration of CD8 T cells, destroying a normal architecture of HFs (**Figure 1A**, right). Interestingly, the haired skin of C3H/HeJ mice with AA also showed linear infiltration of NKG2D-expressing CD8 T cells alongside the border of intact HFs (**Figure 1A**, middle). These features suggest that pathogenic CD8 T cells infiltrate before the onset of hair loss. In flow cytometric analysis, CD8 T cells from both haired and hairless areas express NKGD2 (**Figures 1B, C**). Besides NKG2D, they also expressed TBET and CD103 but not Eomes (**Figure 1D**). These phenotypes are similar to tissue-resident memory (Trm) T cells defined after viral infection in the skin and lung (19–21). In Trm T cells, TBET upregulates the IL-15 receptor, and downregulation of Eomes promotes CD103 expression, enabling their long-term survival and tissue retention, respectively. Therefore, it seems that pathogenic CTLs in AA are tissue-resident memory cells chronically destroying HFs. We also found that NKG2D-expressing CD8 T cells accumulate in the skin draining lymph node (SDLN) but not in the spleen and mLN, suggesting that they have a limited capacity for systemic circulation (**Figure S2**). Interestingly, NKG2D-expressing CD8 T cells were also found in the lung and liver of C3H/HeJ mice with AA. Although its pathological role and clinical significance are unclear, this result suggests that NKG2D-expressing CTLs also distribute outside the skin. Overall, these features show that tissue-resident NKG2D-expressing CTLs accumulate in the lesional and non-lesional skin, lung, and liver in C3H/HeJ mice involved with AA.

### IL-2c Effectively Expands Tregs in the Skin

**Figure 2:**
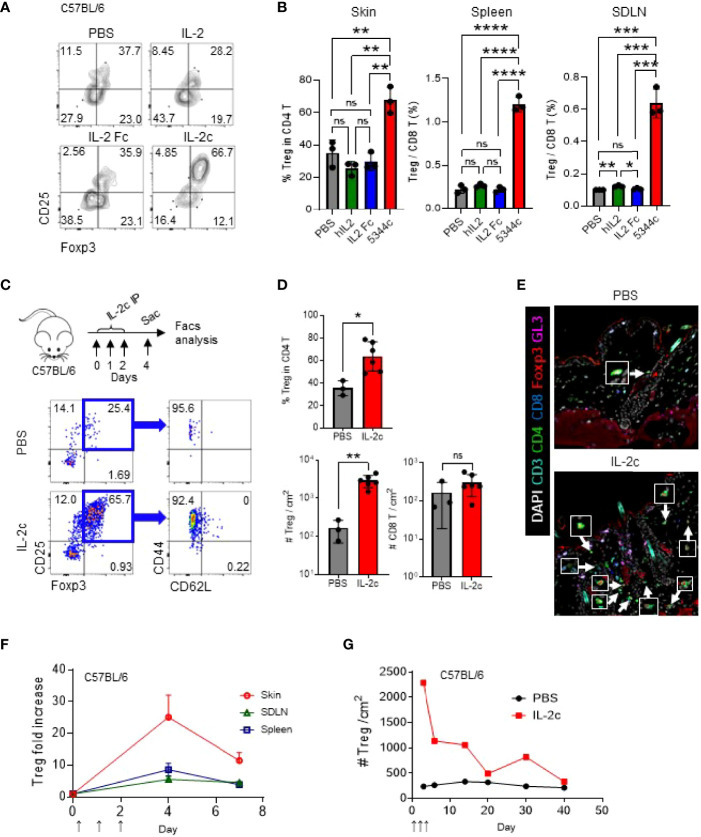
IL-2c effectively expands Tregs in the skin. **(A, B)** Six- to 12-week-old female C57BL/6 mice were intraperitoneally injected with IL-2 (1 μg per 18 g), IL-2 Fc (2.67 μg per 18 g), IL-2c (1 μg of IL-2 and 5 μg of 5344.111 per 18 g) on days 0, 1, and 2, and analyzed on day 4. Representative FACS plots show Tregs in the skin **(A)**, and graphs show quantification of Tregs in indicated organs **(B)**. **(C, D)** Six- to twelve-week-old female C57BL/6 mice were IL-2c injected on days 0, 1, and 2 and analyzed on day 4. Experimental schemes are shown (**C**, top), and representative dot plots show skin Tregs in PBS- or IL-2c-injected mice (**C**, bottom). Graphs show quantification of Tregs in the skin and CD8 T cells in PBS- or IL-2c-injected mice (*n* = 3–6) **(D)**. **(E)** Representative images of skin Tregs of PBS- or IL-2c-injected mice harvested on day 4. Arrows depict Tregs. **(F, G)** Graphs show Treg ratios between IL-2c-injected and non-injected mice in indicated organs **(F)** and kinetics of skin Tregs of PBS- or IL-2c-injected mice **(G)**. Results are from two independent experiments. Horizontal bars indicate mean values, error bars indicate SD, and each dot represents an individual mouse **(B, D)**. An unpaired two-tailed *t*-test was used. *p < 0.05, **p < 0.01, ***p < 0.001, ****p < 0.0001, ns, non-significance (*p* > 0.05).

JAKi could reverse established AA in C3H/HeJ mice and humans (5). However, the patients invariably relapsed upon the cessation of JAKi administration (22). We tested this in C3H/HeJ mice with AA by applying ruxolitinib for 12 weeks and checking them after 10 weeks of treatment endpoint (**Figure S3**). We found that the mice relapsed after treatment cessation, indicating that the treatment effect of JAKi is transient. To test whether expanded Tregs could reverse established AA, we used IL-2, a potent stimulator of Tregs. A previous study showed that three consecutive injections (D0, D1, and D2) of IL-2c (1 μg of mIL-2 plus 5 μg of JES6-1A12) expanded Tregs most efficiently at D4 or D5 in the spleen (23). We found that three consecutive injections are more efficient than one or two injections in the skin, SDLN, and spleen (**Figures S4A, B**). However, prolonged daily injections of IL-2c up to 9 days did not overtly increase the Treg/CD8 T cell ratio in blood after day 5 (**Figure S4**). Next, we compared the effects of equal amounts of IL-2 (1 μg) between pure IL-2, IL-2 Fc, and IL-2c for the expansion of Tregs (**Figures 2A, B**). With three consecutive injections of each reagent containing 1 μg of IL-2, only IL-2c can effectively augment Tregs in the skin, spleen, and SDLN (**Figures 2A, B**). IL-2c-injected mice had more than nine times the number of Tregs compared to control mice, while CD8 T cells were not significantly affected (**Figures 2C, D**). We stained Foxp3 and other T-cell markers in the tissue section and observed that expanded Tregs are located between HFs in the skin (**Figure 2E**). After IL-2c injection, the increased Tregs gradually decreased with a half-life of about a week, and their numbers were normalized after 3 weeks (**Figures 2F, G**).

**Figure 3:**
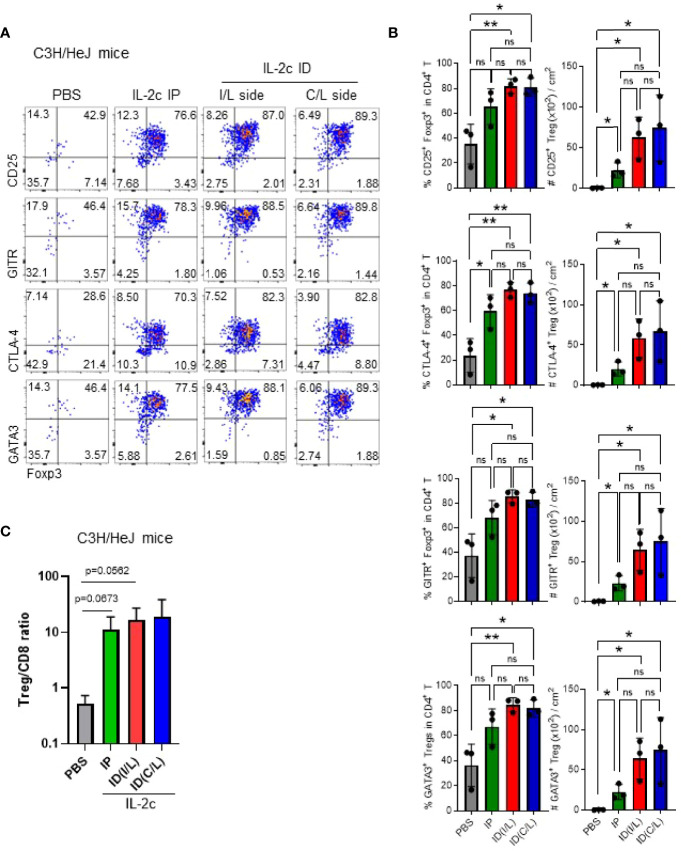
Intradermal injection of IL-2c efficiently expands Tregs in C3H/HeJ mice. Female C3H/HeJ mice were intraperitoneally or intradermally injected with IL-2c on days 0, 1, and 2 and analyzed on day 4. **(A)** Representative dot plots show skin Tregs among total CD4 T cells pre-gated on live CD45.2^+^CD11b¯B220¯TCRβ^+^CD8¯ cells. **(B)** Graphs show the frequencies and absolute numbers of Tregs expressing indicated markers in each group (*n* = 3). Horizontal bars indicate mean values, error bars indicate SD, and each dot represents an individual mouse. An unpaired two-tailed *t*-test was used. *p < 0.05, **p < 0.01. ns, non-significance (*p* > 0.05).

We additionally tested whether ID injection of IL-2c is more effective than IP injection for the expansion of Tregs in the skin (**Figure 3**). Although there was no statistical significance, we found that, compared to IP injection, ID injection induced the expansion of the Tregs more stably, in both ipsilateral and contralateral injection sides. Furthermore, expanded Tregs in the skin maintained the expression of CD25, CTLA-4, GITR, and GATA-3 in C3H mice (**Figures 3A, B**). CD25, CTLA-4, and GITR were also expressed in B6 mice (**Figure S5**), and GATA-3 is a known marker of skin-resident Tregs in B6 mice (24), showing no strain-specific differences. We also confirmed the selective expansion of Tregs in C3H mice by an abrupt increase of CD8 T/Treg ratio upon IL-2c injection (**Figure 3C**). Overall, these results show that ID injection of IL-2c is a favorable method for Treg expansion in the skin of B6 and C3H/HeJ mice.

### Expanded Skin Tregs by IL-2c Express Jagged1, But Does Not Induce Anagen

**Figure 4:**
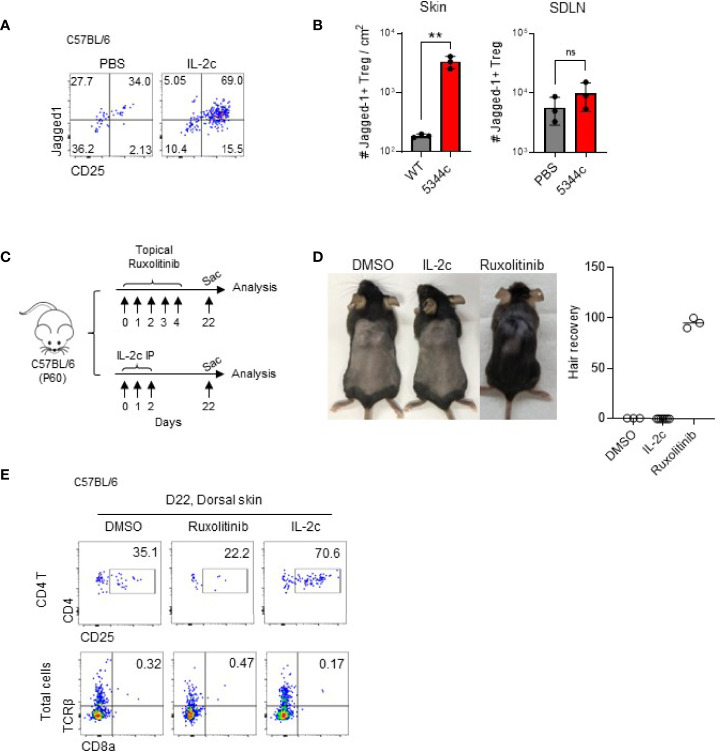
Jagged1^+^ expanded Tregs do not induce anagen. **(A, B)** Six- to 12-week-old female C57BL/6 mice were intraperitoneally injected with IL-2c on days 0, 1, and 2 and analyzed on day 4. Representative dot plots show Jagged1 expression in Foxp3^+^ CD4 T cells **(A)**. Graphs show quantifications of Jagged1^+^ skin Tregs in PBS- and IL-2c-injected mice (*n* = 3) **(B)**. **(C, D)** The experimental scheme shows that 60-day-old female C57BL/6 mice were shaved and topically treated with DMSO or ruxolitinib, or intraperitoneally injected with IL-2c. Mice were analyzed 22 days after treatment **(C)**. Representative photos show hair growth in each group **(D)**. The graph shows the percentages of hair recovery in shaved skin (*n* = 3–6). **(E)** Representative dot plots show frequencies of Tregs and CD8 T cells in the indicated group of mice. Results are from two independent experiments. Horizontal bars indicate mean values, error bars indicate SD, and each dot represents an individual mouse. An unpaired two-tailed *t*-test was used. **p < 0.01; ns, non significance (*p* > 0.05).

Tregs in the skin, but not in SDLN, express Jagged 1, and maintained their expressions after IL-2c treatment (**Figures 4A, B**). A previous study reported that Tregs expressing Jagged1 are required for the telogen-to-anagen transition of HF stem cells (15). To further address the issues of whether the expanded Tregs would facilitate anagen induction, we compared hair growth in mice treated with DMSO, IL-2c, and ruxolitinib at the early telogen phase after shaving (**Figures 4C, D**). As shown previously (7), ruxolitinib induced anagen by day 82, but IL-2c had no effect until day 90 when normal anagen transition occurs. Ruxolitinib is a known negative regulator of Tregs (25), and consistent with this, we found that the frequency of Tregs among CD4 T cells decreased after its treatment (**Figure 4E**). Therefore, it seems like the anagen induction of ruxolitinib is independent of Tregs, and Tregs expressing Jagged 1 are required but not sufficient for the anagen induction in WT mice.

### Expanded Tregs by IL-2c Do Not Reverse Established AA

**Figure 5:**
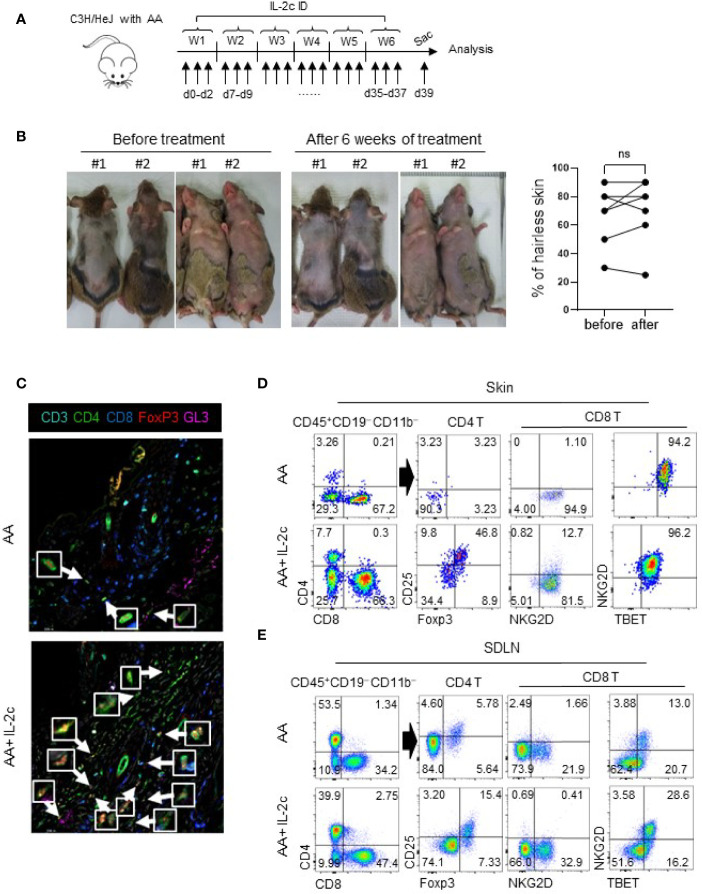
Expanded Tregs by IL-2c do not reverse established AA. **(A)** The experimental scheme shows that C3H/HeJ mice with AA were intradermally injected with IL-2c three times a week for 6 weeks and sacrificed on day 39. **(B)** Representative photos show before and after treatment. The graph shows portions of the hairless area before and after treatment (*n* = 6). **(C)** Representative images show skin Tregs of AA involved C3H/HeJ mice with or without IL-2c injections. Arrows depict Tregs. **(D, E)** Representative dot plots show Tregs and CD8 T cells in the skin and SDLN of AA mice with or without IL-2c injections. Results are pooled from three independent experiments. ns, non significance (*p* > 0.05).

Finally, we tested whether expanded Tregs inhibit autoreactive T cells in AA upon ID injection of IL-2c. Based on the expansion kinetics of Tregs in the skin after IL-2c injection (**Figures 2F**, **S4**), we ID injected IL-2c three times a week for 6 weeks in C3H/HeJ mice with AA as indicated in **Figure 5A**. However, upon completing treatment for 6 weeks, we found no difference in the hairless skin area before and after treatment (**Figure 5B**). In IL-2c-treated mice, imaging analysis revealed expanded Tregs around the HFs (**Figure 5C**). Flow cytometric analysis showed a more than tenfold increase of Tregs among total CD4 T cells in the skin (**Figure 5D**). The fold ratio of CD8 T cells over the Tregs was 320 without IL-2c injections, which decreased to 15 with IL-2c treatment. Therefore, IL-2c selectively induced Tregs in the skin. However, CD8 T cells were still present around the HFs (**Figure 5C**). In addition, flow cytometric analysis showed that NKG2D- and TBET-expressing CD8 T cells are persistent in the skin and SDLN of IL-2c-treated mice (**Figures 5D, E**). Overall, these results indicate that Treg expansion in the skin with IL-2c is not sufficient for the reversal of established AA.

## Discussion

In this report, we used the C3H/HeJ mouse model of AA and showed that infiltrated T cells in the skin have the phenotype of Trm T cells. Using this mouse, we further tested whether expanded Tregs using IL-2c can promote hair growth by inducing anagen and suppressing autoreactive T cells in the skin. Among various modalities to expand Tregs, ID injections of IL-2c could selectively expand skin Tregs by about 8- to 10-fold (**Figure 2**). In addition, expanded Tregs expressed Jagged1 (**Figure 4A**), required for anagen transition of HF stem cells (5). Based on these findings, we expect that IL-2c administration can reverse AA by re-establishing the immune privilege around the HFs and inducing telogen-to-anagen transition. However, contrary to our expectations, expanded Jagged1^+^ skin Tregs neither induced anagen (**Figure 4**) nor reversed established AA lesion by suppressing pathogenic CTLs (**Figure 5**).

In human patients and the C3H/HeJ mouse model of AA, the number of Tregs in the lesion and blood is significantly reduced (26, 27). In addition, compared to healthy control, the IL-10 or TGF-β secretion ability of Tregs in AA showed no significant difference, but the ability to suppress the proliferation of pathogenic T cells is impaired (28). Co-transfer of pathogenic CD8 T or CD25^−^ CD4 T cells with Tregs attenuated the development of AA (29), and depletion of Tregs using anti-FR4 antibody accelerated hair loss in the mouse model of AA (30). Overall, these results indicate that Tregs have a critical role in regulating AA. Based on this, we further tested whether the ID transfer of *ex vivo* expanded Tregs followed by IL-2c administration can boost overall Treg numbers (**Figure S6**). This strategy, however, does not increase the efficiency of IL-2c that effectively expanded endogenous Tregs in the skin.

IL-2 receptor is composed of either IL-2Rα (CD25), IL-2Rβ (CD122), and IL-2Rγ (CD132) on Tregs or IL-2Rβ and IL-2Rγ on effector T cells. Due to IL-2Rα (CD25) on Tregs, they have a 100-fold higher affinity to IL-2 than effector T cells (31). Because of this, low-dose IL-2 can selectively stimulate Tregs, which are attractive therapeutic modalities in several autoimmune or inflammatory diseases (14, 32). In human patients and mouse models with AA, defective Treg numbers and functions have been proposed, and a small-scale clinical trial suggested that low-dose IL-2 would have therapeutic effects in human patients (33). However, a multicenter prospective placebo-controlled study failed to show the significant therapeutic advantages of low-dose IL-2 in AA (34). This study showed that low-dose IL-2 could expand naïve Tregs for up to 12 months after IL-2 administration. However, Tregs with memory phenotype remained unaffected, suggesting that specific Treg populations could be critical for re-establishing tolerance. One caveat of using low-dose IL-2 treatment is determining its optimal dose for each individual because its slight change can activate STAT5 signaling on conventional memory T cells and NK cells (35). Also, due to the short half-life of IL-2, patients need to be administrated frequently at close intervals (36).

To overcome the issues of using pure IL-2, we used IL-2c, a complex of IL-2 and anti-IL-2 antibodies. IL-2c has dramatically extended the *in vivo* half-life of IL-2 and can preferentially stimulate effector T cells or Tregs dependent on the tertiary structure of the complex (37–41). These properties are applicable for tumor immunotherapy and inflammatory diseases, respectively. For example, selectively expanded Tregs by IL-2c showed promising results in allograft survival, prevention of arthritis, experimental autoimmune encephalomyelitis (EAE), graft-versus-host disease (GvHD), and T1D (42, 43) (23, 44). The previous report used anti-human IL-2 (F5111) or anti-mouse IL-2 (JES6-1A12) antibodies and showed their therapeutic effect in diabetic NOD mice (44). In this report, we used an anti-human IL-2 antibody (BD Bioscience, clone 5344.111), which has a similar effect of Treg expansion compared to JES6-1A12. However, we found that expanded Tregs in the skin neither induce anagen nor reverse established AA.

Another form of engineered IL-2 is IL-2 mutein (Fc.IL-2), which fused murine IgG2a Fcv with IL-2 mutated on the CD122 binding site (45). It has increased *in vivo* half-life (79.7 h) and requires CD25 for efficient receptor binding, which induces long-term expansion of Tregs, preventing T1D onset in NOD mice. They showed that IL-2 mutein has a better *in vivo* efficacy to expand Tregs than IL-2c in a single injection scheme. However, we found that three consecutive injections are better than a single injection (**Figures S4A, B**), and mutant proteins have the issue of immunological rejection with repetitive usage. Therefore, comparing the *in vivo* efficiencies of IL-2c and IL-2 muteins requires further investigation.

In this study, we used female C3H/HeJ mice whose 30%–90% of the entire skin is affected by AA (**Figure 5**) to mimic the condition of human patients with alopecia totalis (AT) or alopecia universalis (AU) . It is possible that early treatment of IL-2c before the full establishment of AA in C3H/HeJ mice might inhibit disease progression. Combination therapy with depletion of CD8 T cells or other chemicals that can deplete Trm T cells in inflamed tissues (e.g., JAKi) would be worthy of testing for future therapeutic potential. Overall, our study suggests the need for combination treatment other than IL-2c for AA.

## Data Availability Statement

The original contributions presented in the study are included in the article/**Supplementary Material**. Further inquiries can be directed to the corresponding author.

## Ethics Statement

The animal study was reviewed and approved by the Institutional Animal Care and Use Committee of POSTECH (2013–01–0012).

## Author Contributions

EL designed and performed experiments, analyzed data, and wrote the draft. MK performed immunofluorescence. YL supervised the study, analyzed data, and wrote the manuscript. All authors contributed to the article and approved the submitted version.

## Funding

This work was supported by the New Faculty Startup Fund from Seoul National University (370C-20210072) and the Korean Ministry of Science, Information/Communication Technology, and Future Planning (2021R1F1A1054395, 2022R1A2C1007692).

## Conflict of Interest

The authors declare that the research was conducted in the absence of any commercial or financial relationships that could be construed as a potential conflict of interest.

## Publisher’s Note

All claims expressed in this article are solely those of the authors and do not necessarily represent those of their affiliated organizations, or those of the publisher, the editors and the reviewers. Any product that may be evaluated in this article, or claim that may be made by its manufacturer, is not guaranteed or endorsed by the publisher.
