# Supplementary figures and images for: Selective Expansion of Tregs Using the IL-2 Cytokine Antibody Complex Does Not Reverse Established Alopecia Areata in C3H/HeJ Mice
Source: Front Immunol. 2022 Jun 15;13:874778. doi: 10.3389/fimmu.2022.874778 (PMC9254621; doi:10.3389/fimmu.2022.874778)

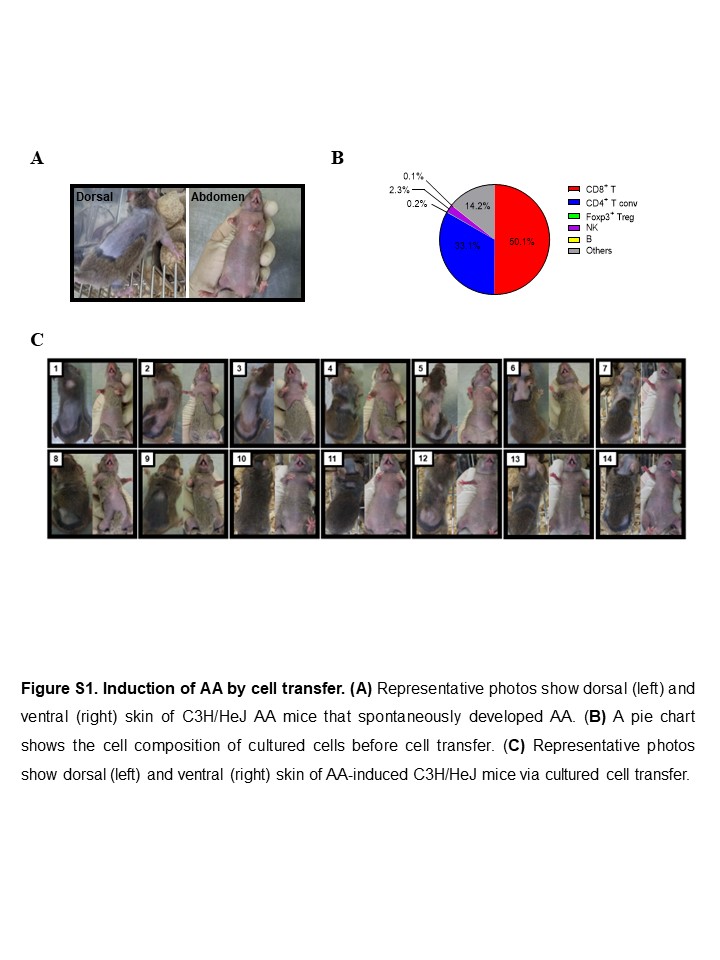

Supplement: Supplementary file 1 [file Image_1.jpeg]

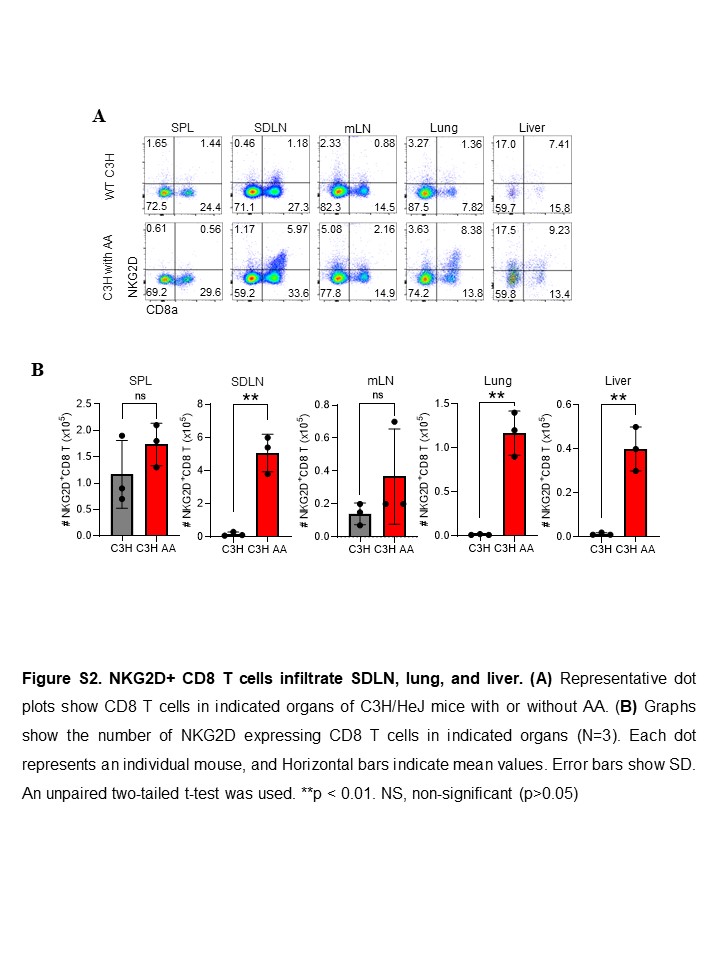

Supplement: Supplementary file 2 [file Image_2.jpeg]

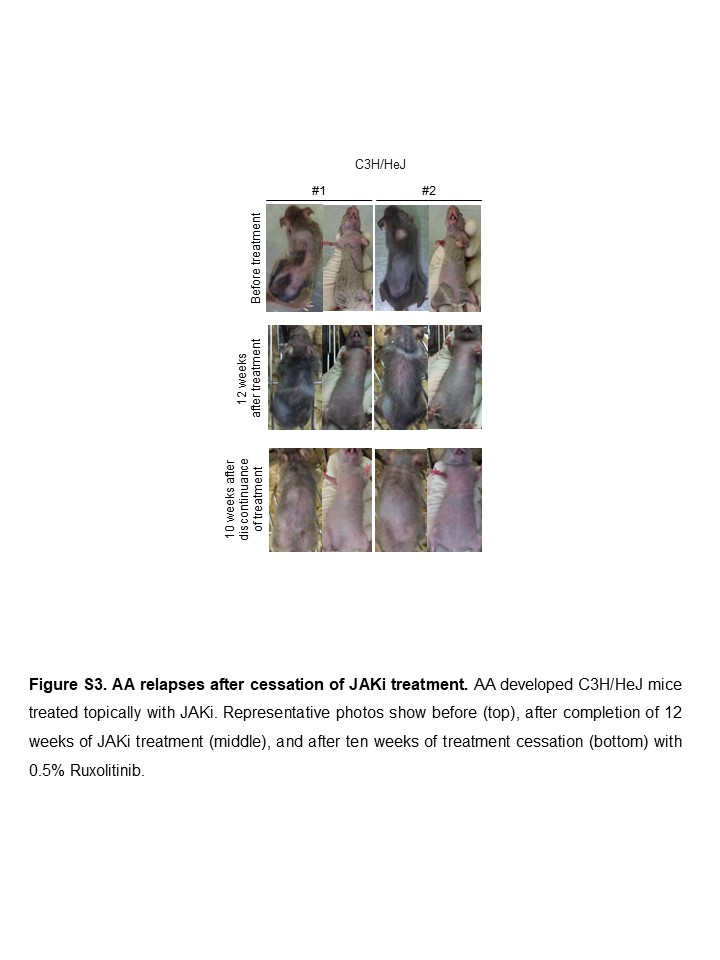

Supplement: Supplementary file 3 [file Image_3.jpeg]

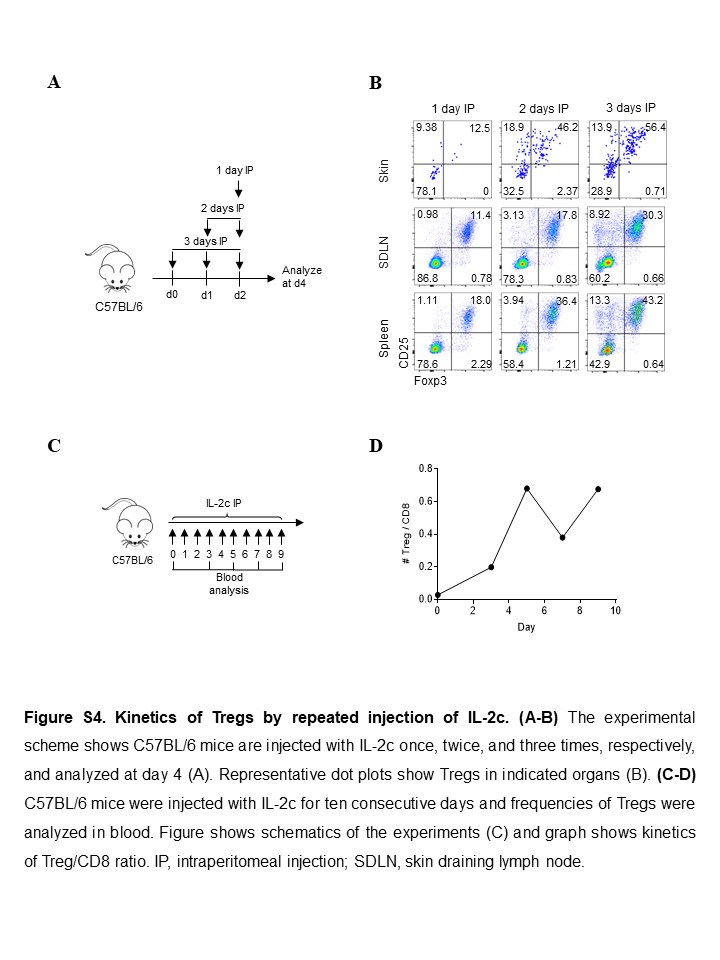

Supplement: Supplementary file 4 [file Image_4.jpeg]

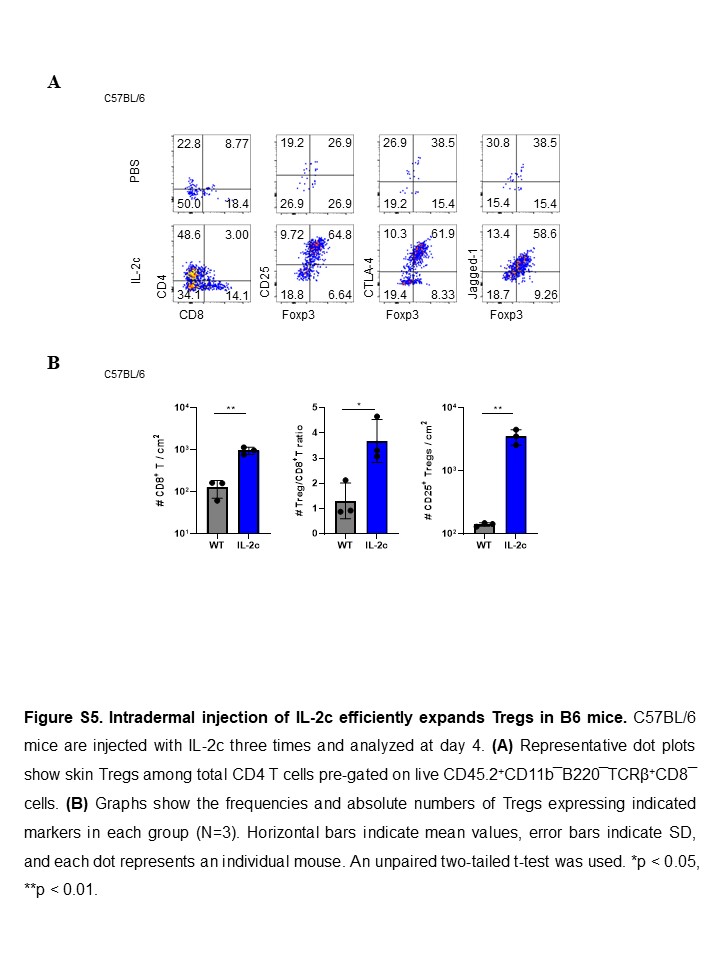

Supplement: Supplementary file 5 [file Image_5.jpeg]

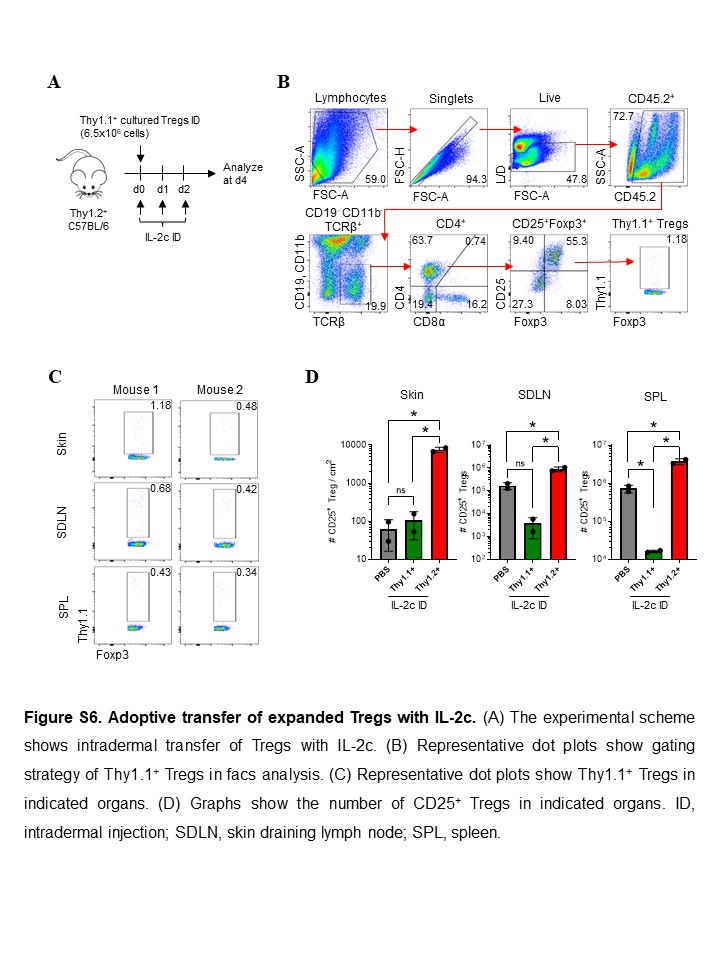

Supplement: Supplementary file 6 [file Image_6.jpeg]
